# The Impact of Spaceflight and Simulated Microgravity on Cell Adhesion

**DOI:** 10.3390/ijms21093031

**Published:** 2020-04-25

**Authors:** Xiao Lin, Kewen Zhang, Daixu Wei, Ye Tian, Yongguang Gao, Zhihao Chen, Airong Qian

**Affiliations:** 1 Lab for Bone Metabolism, Key Lab for Space Biosciences and Biotechnology, School of Life Sciences, Northwestern Polytechnical University, Xi’an, 710072, China; linxiao@nwpu.edu.cn (X.L.); zkw@mail.nwpu.edu.cn (K.Z.); tianye@nwpu.edu.cn (Y.T.); gaoyongguang@nwpu.edu.cn (Y.G.); chzhh@mail.nwpu.edu.cn (Z.C.); 2 Xi’an Key Laboratory of Special Medicine and Health Engineering, School of Life Sciences, Northwestern Polytechnical University, Xi’an 710072, China; 3 Research Center for Special Medicine and Health Systems Engineering, School of Life Sciences, Northwestern Polytechnical University, Xi’an 710072, China; 4 NPU-UAB Joint Laboratory for Bone Metabolism, School of Life Sciences, Northwestern Polytechnical University, Xi’an 710072, China; 5 Key Laboratory of Resource Biology and Biotechnology in Western China, Ministry of Education, School of Medicine, Northwest University, 229 Taibai North Road, Xi’an 710069, China; weidaixu@nwu.edu.cn

**Keywords:** spaceflight, simulated microgravity, cell adhesion, adhesion molecules, cytoskeleton

## Abstract

Microgravity induces a number of significant physiological changes in the cardiovascular, nervous, immune systems, as well as the bone tissue of astronauts. Changes in cell adhesion properties are one aspect affected during long-term spaceflights in mammalian cells. Cellular adhesion behaviors can be divided into cell–cell and cell–matrix adhesion. These behaviors trigger cell–cell recognition, conjugation, migration, cytoskeletal rearrangement, and signal transduction. Cellular adhesion molecule (CAM) is a general term for macromolecules that mediate the contact and binding between cells or between cells and the extracellular matrix (ECM). In this review, we summarize the four major classes of adhesion molecules that regulate cell adhesion, including integrins, immunoglobulin superfamily (Ig-SF), cadherins, and selectin. Moreover, we discuss the effects of spaceflight and simulated microgravity on the adhesion of endothelial cells, immune cells, tumor cells, stem cells, osteoblasts, muscle cells, and other types of cells. Further studies on the effects of microgravity on cell adhesion and the corresponding physiological behaviors may help increase the safety and improve the health of astronauts in space.

## 1. Introduction

Microgravity is a state in which the gravitational force acting in any single direction is negligible. True microgravity is present during parabolic flights, on orbiting spacecraft, or space labs on the international space station (ISS). However, the absence of gravity in space (zero gravity) can also be simulated as microgravity on earth, for which many ground-based tools were developed. These include the clinostat, magnetic levitation, and the rotating wall vessel bioreactor [1,2]. Recently, researchers are investigating the effects of microgravity on the physiological state of cells and the underlying mechanisms using earth-based simulation techniques.

The biological effects of weightlessness on the human body have been recognized and investigated since the earliest space flights, forming one of the most important research directions in the field of aerospace medicine. During both short- and long-term space flight, astronauts are exposed to several risk factors related to radiation and the absence of gravity. Weightlessness eliminates the normal hydrostatic pressure gradient within the body, leading to cardiovascular dysfunction, and also induces muscle atrophy, bone loss, as well a depression of immune function [3].

Cell adhesion is a prerequisite for multicellularity in both evolution and individual development, providing a basis for the integrity of organisms [4]. The spatial arrangement of cells mediated by adhesion plays important roles in physiological states such as tissue morphogenesis and wound healing, as well as the progression of pathological conditions such as cancer [5,6,7]. Biological tissues are primarily remodeled through the interaction of cells with their microenvironment via adhesion molecules, but also through the secretion and adsorption of soluble factors [8]. Thus, cell adhesion also plays an important role in the physiological changes observed under microgravity [9]. Many different cell adhesion molecules, which are transmembrane proteins, regulate homophile (cell–cell adhesion) and homophile binding (cell–matrix adhesion) of cells [4].

In this review, we introduce the biological functions of cell adhesion and summarize the roles of the four major classes of cellular adhesion molecules (CAMs) on cell adhesion, namely the integrins, immunoglobulin superfamily (Ig-SF) proteins, cadherins, and selectin in regulating cell adhesion. Furthermore, the effects of microgravity on the adhesion of endothelial cells, immune cells, cancer cells, stem cells, osteoblasts, muscle cells, and other types of cells both during spaceflight and under simulated conditions, are reviewed.

## 2. The Biological Functions of Cell Adhesion

Physiological processes such as embryonic development and tissue homeostasis, as well as pathological conditions such as cancer, rely on the temporal and spatial control of cell adhesion [8]. Adhesion allows cells to transfer forces, sense their surrounding microenvironment, and coordinate their biological behaviors [10]. Both cell–cell and cell–ECM adhesion have unique components, but both are directly linked to the cell’s actin cytoskeleton [11]. Thus, mechanical and/or chemical stimuli are transferred and integrated to induce or control cell proliferation, migration, and differentiation.

Intercellular adhesion is among the most important aspects. For example, cell–cell adhesion within the vessel wall controls various dynamic processes of endothelial cells, including angiogenesis and vascular remodeling [12]. The observation that vascular endothelial cadherin (VE-cadherin) knockout is embryonically lethal in mice because of defects in blood vessel pattern formation demonstrated the importance of VE-cadherin-based adherens junctions in maintaining the function of the vascular barrier [13]. Cell–cell contacts also crucially regulate cell survival, and in human keratinocytes, they constitute the main mechanism that controls proliferation, which is regulated by the junctional protein plakoglobin [14]. Similarly, apoptosis of granulosa cells is prevented by cell contact mediated by N-cadherin molecules on the surface membranes of adjacent cells [15]. In addition, cell migration is essential for embryonic morphogenesis, tissue remodeling and wound healing in adults, as well as pathological cancer cell migration. Intercellular adhesion promotes cancer cell mobility by increasing the polarization of individual cells, which plays an important role in the pathogenesis of tumors [16]. The dynamic regulation of cell–cell adhesion in neural crest cells is crucial for maintaining their migration capacity, which allows them to maintain tissue integrity [17]. In the process of wound healing, sheets of adhering keratinocytes can promote the migration of these cells into the wound [18].

In addition to the adhesion between cells, their adhesion to the extracellular matrix (ECM) via cell adhesion molecules is also crucial [19]. To migrate over the matrix, the cells first extend filopodia and lamellipodia, forming focal adhesions, and finally cytoplasmic tails are then retracted on opposite ends. Active complexes of ligands such as integrins promote the rearrangement of the cytoskeleton and activate intracellular signaling cascades. The events downstream of integrins, such as activation of focal adhesion kinase (FAK) and Src, or the recruitment of adaptor proteins such as zyxin and vinculin, depend on ligand binding and receptor clustering [20]. In a process known as cytoskeletal strengthening integrin-mediated cell–ECM adhesion is enhanced through interactions with cytoskeletal proteins, among which vinculin is important in stimulating actin polymerization and recruiting actin remodeling proteins. Both cell–matrix and cell–cell adhesion is significantly impaired when vinculin is removed [21]. Furthermore, the mechanical elasticity or stiffness of the ECM profoundly influences cell behavior. The mechanical cues of the ECM are sensed by yes-associated protein (YAP)/TAZ to activate specific gene expression and cell behaviors [22].

## 3. Role of Major Adhesion Molecules in Cell Adhesion

CAM is a general term for macromolecules responsible for the contact and binding between cells or between cells and ECM through receptor-ligand interactions. CAMs participate in many different physiological and pathological processes including inflammation, the immune response, blood coagulation, wound healing and tumor metastasis [23]. Here, we will introduce the four major classes of adhesion molecules that regulate cell adhesion: the integrins, Ig-SF proteins, cadherins, and selectin (Table 1).

### 3.1. Integrins

Integrins are a large family of type I transmembrane glycoproteins found in all animal lineages. They are non-covalently-linked αβ-heterodimeric receptors with a short cytoplasmic domain and a large extracellular domain. Mammalian cells possess integrins with 18 α-subunits (α_1–11_, α_V_, α_IIb_, α_L_, α_M_, α_X_, α_D_ and α_E_) and eight β-subunits (β_1–8_), which are assembled into 24 known αβ-heterodimers [35]. Integrins are critical for cell adhesion, polarity, migration, as well as the formation and maintenance of normal tissue structure. Activated integrins play a pivotal role by forming a connection between the ECM and the actin cytoskeleton to facilitate cell adhesion [24]. Integrin-mediated adhesions beneath the lamellipodia initially form focal complexes, which further mature into longer lived and elongated focal adhesions. They serve as anchors for actin stress fibers, which in turn exert a force on the Fas to which they are connected [36]. In addition, the adhesion is mediated by several factors, including specific conformational affinity of integrin receptors to their cognate extracellular ligands, the mechanical force acting on the point of adhesion, the intracellular partners that bind integrins into discrete adhesive structures, and intracellular trafficking of integrins [37].

### 3.2. Immunoglobulin Superfamily

Ig-SF comprises the largest and most diverse class of cell adhesion molecules. The members of the Ig-SF are characterized by the presence of at least one Ig-like extracellular domain encompassing 70–110 amino acids that mediate calcium-independent cell adhesion [38]. Important examples of Ig-SF proteins include intercellular adhesion molecule (ICAM), vascular cell adhesion molecule (VCAM), and platelet endothelial cell adhesion molecule (PECAM). The adhesion mediated by these molecules is critical for normal immunological recognition, inflammation, and signal transduction, as well as pathological processes such as tumor metastasis.

#### 3.2.1. ICAM-1

ICAM-1 is mainly expressed in epithelial cells, endothelial cells, leukocytes, and neutrophils. It contains five Ig-like domains and binds to β_2_ integrin molecules present on leukocytes, as well as MAC-1 (CD11b/CD18), and LFA-1 (CD11a/CD18) [39]. ICAM-1 mainly mediates cell–cell adhesion by binding to specific ligands in the ECM and surrounding cells [25]. It participates in the trafficking of inflammatory cells, in cell–cell interaction during antigen presentation, in signal transduction cascades through outside-in signaling, and in certain aspects of microbial pathogenesis [39]. Goh et al. demonstrated that artificial overexpression of ICAM-1 in myoblasts increases their mutual adhesion as well as the formation and size of myotubes [40]. Guo et al. reported that ICAM-1 is a crucial regulator of adhesion between endothelial progenitor cells (EPCs) and mesenchymal stem cells (MSCs) through p38/MAPK signaling pathway, it is of important for neovascularization and artificial bone regeneration [41]. Moreover, the enhanced expression of ICAM-1 in cancer cells is sufficient to mediate the adhesion of immune cells, which is correlated with poor prognosis [42]. These evidence prove that ICAM-1 plays an important role in cell adhesion.

#### 3.2.2. VCAM-1

VCAM-1, also known as CD106, is a 90-kDa glycoprotein that is predominantly expressed by endothelial cells. Its expression is activated by ROS, oxidized low-density lipoprotein, high glucose concentrations, toll-like receptor agonists, shear stress, and pro-inflammatory cytokines such as TNFα [43]. Ligands of VCAM-1 include integrins α_4_β_1_, α_4_β_7_, α_M_β_2_, α_9_β_1_, and α_D_β_2_, which mediate firm adhesion among cells [44].

Because of the function of VCAM-1 in cell adhesion, it is essential, and a knockdown of VCAM-1 causes embryonic death in mice [45]. In vascular endothelial cells, inhibiting VCAM-1 expression has an anti-inflammatory effect by attenuating leukocyte adhesion to endothelial cells, and stimulated expression of VCAM-1 leads to increased adhesion of monocyte to endothelial cells. p38 MAP kinase and NF-κB signaling pathways might take part in regulating VCAM-1 expression [26,27]. In addition to endothelial cells, in cancer cells, knockdown of VCAM-1 in MDA231 breast cancer cells inhibited lung metastasis by reducing the adhesion to U937 leukocytes [46], the binding of VCAM-1 to α_4_β_1_ integrin might be responsible for tumor angiogenesis and metastasis [47]. Thus, VCAM-1 mediates the adhesion and tissue invasion of various types of cells.

#### 3.2.3. PECAM-1

PECAM-1, also known as CD31, is a crucial mediator of the adhesion and accumulation of platelets [28]. Hematopoietic cells selectively express PECAM-1, and it is highly enriched at endothelial cell–cell junctions [48]. PECAM-1 contains six extracellular Ig-like domains that mediate the attraction and adhesion of leukocytes to endothelial cells, for example by enhancing eosinophil adhesion to IL-4-stimulated HUVECs in an α_4_β_1_ integrin-dependent manner [49,50]. Additionally, it also contains a transmembrane domain of 19-amino acids and a cytoplasmic tail encompassing 117-amino acid, which contains several phosphorylation sites that are important for intracellular signaling [51]. The cytoplasmic domain of PECAM-1 has two ITIM motifs that can be phosphorylated by Src kinase and recruit β-catenin and SHP-2 in endothelial cells. Since β-catenin is critical for adherent junctions of endothelial cells, PECAM1 can also inhibit the formation of endothelial cells connections [52].

### 3.3. Cadherins

Cadherins are a large superfamily of calcium-dependent transmembrane adhesive proteins that are responsible for cell–cell adhesion in all soft tissues as major adhesive proteins [4]. Extracellular regions of cadherin in a trans orientation mediate the adhesion between neighboring cells, while the cytoplasmic tails of cadherins interact with effector proteins such as catenin, which connects them to the actin cytoskeleton. Numerous physiologically and pathologically processes depend on the ability of cells to sense, transmit, and respond to mechanical forces and trigger intracellular signaling pathways, and these signals are mediated by adhesive complexes formed by cadherins [53].

Among the four main subfamilies, classical cadherins are the most studied and the other three such as desmosomal cadherins, protocadherins, and atypical cadherins are less well studied. Epithelial (E)-, neuronal (N)-, and vascular endothelial (VE)-cadherins derived from epithelial, neuronal, and vascular endothelial cells, respectively, are the prominent classical cadherins [54]. Adhesion mediated by E-cadherins is one of the main regulators of epithelial cell morphology and differentiation. The p120 catenin regulates the stability of E-cadherin, contributing to the maintenance of cell–cell adhesion in epithelial cells [29]. Recently, it was reported that tumor suppression depends on the normal functioning of E-cadherin in epithelial, mainly because of E-cadherin-mediated cell-to-cell adhesions [55]. Accordingly, the lack of E-cadherin aggravates the invasion and metastasis of many epithelial tumors. The full length or 90 kD N-terminal fragment of N-cadherin can promote cell–matrix adhesion [56]. In tumor cells, N-cadherin can promote tumor invasion via intercellular adhesions [30]. Additionally, the reorganization of the cytoskeleton during tumor cell migration and metastasis is regulated through synergistic effects between N-cadherin and fibroblast growth factor receptor (FGFR), which control the balance of intercellular and cell–matrix adhesions [57]. VE-cadherin is a major component of the adhesive connections between endothelial cells. The stability of VE-cadherin mediated adhesions depends on tyrosine phosphorylation of the VE-cadherin–catenin complex [31], which is proved by evidence that increased tyrosine phosphorylation of VE-cadherin and β-catenin is correlated with increased endothelial cell confluence [58]. As a result, classical cadherins mediate the cell–cell and cell–ECM adhesion, which is crucial for tissue morphogenesis and integrity.

### 3.4. Selectin

Selectins are a family of vascular cell adhesion molecules comprising of the three Ca^2+^-dependent lectins: E-selectin, L-selectin, and P-selectin. E-, L-, and P-selectin are expressed on the surface of endothelial cells, leucocytes, and platelets, respectively, and have similar structures encompassing an epidermal growth factor-like domain, a transmembrane domain, a sequence of repeats, and also a cytoplasmic tail [59]. P-selectin mediates the adhesion of platelets to tumor cells. L-selectin can promote the recruitment of marrow-derived cells, and accompanied by E-selectin promotes extravasation of tumor cells. Selectins mediate the interaction between tumor cells through the activation of integrins and secretion of pro-metastatic chemokines in the tumor microenvironment, contributing to metastasis and poor prognosis of cancer patients [32]. Moreover, the selectin–selectin ligand interactions of endothelial cells and tumor cells result in the extravasation of tumor cells [33]. Selectin also participates in constitutive lymphocyte homing. The adhesion and localization of leukocytes on vascular surfaces mediated by selectin is a key stage in the inflammatory process, which is accompanied by worsening of psoriasis, arthritis or asthma when aberrant homing of leukocytes occurs [34]. Therefore, evidence supports that selectins are important for physiological and pathological processes including inflammation, the immune response, and cancer metastasis.

## 4. Effects of Microgravity on Cell Adhesion

### 4.1. Microgravity Regulates Adhesion and Cytoskeleton Arrangement of Endothelial Cells

Microgravity is a stress factor that puts a burden on human cardiovascular physiology and functioning. Under microgravity, endothelial cell-related arterial remodeling occurs, which is a major cause of orthostatic intolerance in astronaut’s return to normal gravity [60]. Endothelial cells can be induced to express adhesion molecules and secrete cytokines as signals for cells in the bloodstream. Gene profile analysis of human umbilical vein endothelial cells (HUVECs) cultured on the ISS revealed that the expression of cell adhesion, such as *VCAM-1*, *ICAM-1*, and *CD44* showed significant changes of expression compared with ground-based control cultures, along with the depolymerization of microtubules and actin filaments in endothelial cells, which is also accompanied by the decreased secretion of pro-angiogenetic and pro-inflammatory cytokines [61,62]. In addition, under simulated microgravity, the expression of adhesion molecules by endothelial cells showed patterns similar to those observed in spaceflight experiments in real microgravity. After incubation in a random positioning machine (RPM) for 35 days, the expression of VCAM-1 and ICAM-1 was increased in endothelial cells [63], which has also been demonstrated by a rat model that expression of E-selectin and VCAM-1 in endothelial cells was elevated in basilar and carotid arteries under hindlimb unweighted (HLU) condition [64]. Therefore, in a ground-based simulated microgravity model, microgravity has the same effect on the expression of adhesion molecules in an endothelial cell in vivo and in vitro.

The expression of the adhesive protein can affect the adhesion ability and cytological changes of endothelial cells under microgravity. With the increased secretion of VCAM-1 and ICAM-1, prolonged microgravity induced tubular structures and the formation of multicellular spheroids formation from endothelial cells. Moreover, decreased expression of fibronectin protein in adherent cells helps to separate cells from monolayers because fibronectin is involved in cell adhesion to culture plates [63]. After exposure to microgravity in the rotary cell culture system (RCCS), there was a decrease in the cell adhesion rate and changes of cytoskeletal structures of HUVECs after return to normal gravity, which resulted in the initiation of cell apoptosis via mechanisms involving mTOR/Apaf-1 and miR-22 signaling. The decreased cell adhesion ability triggers reduced actin fiber formation that might contribute to growth inhibition of endothelial cells [65]. Therefore, in endothelial cells, microgravity exerts important regulatory effects on cell adhesion, actin filament arrangement, and tube formation abilities (Table 2).

### 4.2. Microgravity Regulates Adhesion and Activation of Immune Cells

The lack of gravity during space missions is one of the major stress factors that is responsible for the dysfunction of the inherent and adaptive immune system in astronauts [66]. Cells of the immune system are also sensitive to microgravity. The influence of microgravity on adhesion molecule of immune cells has been extensively studied in space missions. After 11 days on SpaceX CRS-3, primary human M1 macrophages displayed a reduced expression of ICAM-1 compared to controls on the ground [67]. The reduction of gravity-responsive ICAM-1 was activated by mechanically sensitive signals in the cell-polycarbonate binding region, leading to blocked CD4^+^ T lymphocyte activation and immune response. However, different from that, in macrophage-like differentiated human U937 cells, the expression of ICAM-1 was increased during 2D clinostat as well as on the orbital SIMBOX/Shenzhou-8 mission, while had no effect on ICAM-1 in nondifferentiated U937 cells. The changes in gene expression of ICAM-1 have a great influence on their interaction with T lymphocytes [68]. These results showed that the expression trend of adhesion molecules was different in types and differentiation stage of immune cells, and also in different microgravity conditions.

Responding to microgravity, adhesion proteins act as mechanosensors that mainly contributed to the activity, migration, and adhesion of immune cells. For example, Liu et al. reported that the adhesion of monocytes/macrophages to the endothelium of the common carotid artery of HLU rats was enhanced in vivo [69]. Further study demonstrated that the recruitment of monocyte to the aortic endothelium was significantly increased under simulated microgravity, along with NF-κB-mediated increased expression of E-selectin, VCAM-1 and monocyte chemoattractant protein (MCP-1) in the abdominal aorta of rats [70]. In addition, after short-duration spaceflight, the peripheral monocytes from astronauts showed no change in the number of cells but a significantly reduced expression of CD26L and major histocompatibility complex, class II, DR (HLA-DR), which are known regulators of cell adhesion between lymphocyte and endothelial cells [71]. Consistent with this finding, the ability of peripheral blood mononuclear cells to adhere to the ICAM-1 substrate was reduced under simulated microgravity and shear flow conditions in vitro [72]. Although the force from the blood flow plays a decisive role in the monocyte’s adhesion cascade and initial capture with endothelial by selectins [73], the adhesion ability of immune cells and endothelial cells can also be impaired in microgravity. Therefore, it can be speculated that microgravity can regulate the adhesion of immune cells to endothelial cells by regulating the expression of adhesion molecules, which may have an important effect on the activity of immune cells (Table 3).

### 4.3. Microgravity Inhibits Tumor Cells Adhesion

Microgravity was reported to affect the focal adhesions, cytoskeletal arrangement, and adhesion of cancer cells, which in turn affects their migration, proliferation, and metastasis. Focal adhesion is one of the mechanosensitive structures. Tumor cells are sensitive to mechanical forces through focal adhesion, leading to stress-dependent adhesion in the microgravity environment. In microgravity, the formation of focal adhesions (paxillin and vinculin) in BL6-10 melanoma cells was reduced, while FAK was recruited and activated, which led to enhanced apoptosis via FAK/RhoA-regulated mTORC1 and AMPK signaling, together with a reduction of proliferation and metastasis compared with control cells at normal gravity [74,75]. Consistent with with the findings in melanoma cells, microgravity was found to regulate the focal adhesions and associated signaling molecules in glioma cells (U251 cells), such as FAK and RhoA/Rock [76]. Moreover, in malignant human MCF-7 cells, the number and clustering of focal adhesions were reduced under simulated microgravity, along with downregulation of β_1_ and β_4_ integrin and FAK activity. [77]. However, in prostate cancer cells, the expression of *Talin1*, *Vinculin*, and *Cdh1* involved in focal adhesions were upregulated in multicellular spheroids of cells when exposed to simulated microgravity [78]. Thus, as the component of focal adhesions, Vinculin, FAK, and RhoA are the most important molecules that transmit external integrin-mediated signals into the cell, the biochemical pathways, and functions of tumor cells could be influenced by the disruption of adhesion structures.

Similarly, cytoskeleton as another mechanically sensitive structure also participate in tumor cell adhesion. For example, in adherent human lung cancer cells (cell line CRL-5889), they showed attenuated adhesion with a spherical arrangement of the actin filaments under simulated microgravity, and with accelerated apoptosis [79]. In addition, along with reduced focal adhesions, the microfilaments and microtubules were disrupted in MCF-7 cells, resulting in reduced cell migration [77]. Consistent with this, Nassef et al. demonstrated that the F-actin and tubulin were rearranged and the filopodia- and lamellipodia-like structures were appeared during early microgravity, accompanied with decreased expression of E-cadherin and β_1_ integrin in MCF-7 cells [80]. The expression of cell junction protein E-cadherin was reduced through the regulation of the E-cadherin autodegradation pathway, leading to multicellular spheroids formation of MCF-7 cells [81]. Taken together, during microgravity exposure, the synergistic effect of cytoskeleton and E-cadherin is involved in the tumor cell adhesion and multicellular spheroids formation process, which might eventually lead to tumorigenesis.

The effects of real and ground-based simulated microgravity on the expression and structure of the adhesion molecule in cancer cells are different. Kopp and colleagues demonstrated that real microgravity induced the expression of *VCAM1* and *ICAM1* in thyroid cancer cells (FTC-133 cell line) during sounding rocket flight [82]. However, ground-based studies revealed that the expression of ICAM1 protein was lower in RPM-induced thyroid cancer cells. Moreover, focal adhesion factor cofilin has different performance in real and simulated microgravity. Real microgravity elevated the expression of F-actin-binding protein cofilin, while the expression of cofilin was reduced in the RPM sample with disorganized vinculin structure [82]. Due to the link between the ECM and the cytoskeleton, the different expression patterns of VCAM1, ICAM1, and cofilin might influence cell adhesion in real and simulated microgravity through regulating actin cytoskeleton dynamics. Moreover, in MDA-MB-231 cells, the protein expression of ICAM1 and VCAM-1 was increased after the 31st parabolic flight. However, there was no expression change of ICAM1 and VCAM1 in cells under RPM-exposure [83]. These results may indirectly reflect the different biological effects of real and simulated microgravity. Further studies are still needed to fully reveal the different dynamic molecule changes of tumor cell adhesion in real and simulated microgravity at the single-molecule level (Table 4).

### 4.4. Microgravity Regulates Adhesion and Fate Determination of Stem Cells

Stem cells are a type of cells with self-renewal capacity and multi-directional differentiation potential. Microgravity has been shown to provide important signals for the fate of stem cells. The adhesion ability and expression of related genes in many stem cells were also observed to change under microgravity. Ratushnyy et al. reported that a total of 84 focal adhesion genes were identified as differentially expressed in multipotent mesenchymal stromal cells after 96 h of simulated microgravity [84]. Among them, the expression of *ITGA11*, *ITGAV*, *ITGB1*, *DOCK1*, *ROCK2*, *PTEN*, *FAK*, *ARHGAP5*, and *AKT3* was downregulated [84]. These are genes that are related to the cytoskeleton and might be a reason for the reduced adhesion of MSCs in microgravity.

Most of the literature supports the relation between adhesion molecule expression and cytoskeletal structure and adhesion ability of the cell. With the increasing time under simulated microgravity, the number of VCAM-1-positive human MSCs (hMSCs) and the expression of ICAM-1 were decreased. Moreover, the number of hMSCs with a disordered actin cytoskeleton gradually increased and the cells showed a contracted phenotype [85,86]. Besides, in rat MSCs on board the SJ-10 Satellite, the expression of *VCAM1*, *ICAM1*, *CD44*, and *vinculin* was reduced along with depolymerization of actin filaments and the accumulation of microtubules due to true weightlessness in space [87]. With experiments in vitro and in vivo, it was demonstrated that redistribution of vinculin also occurred in hMSCs after 6 h of simulated microgravity [85], and the number of vinculin-containing focal adhesions was decreased in rat MSCs after 28 days of HLU [88]. Therefore, compared with conventional culture conditions, microgravity causes a part of stem cells to show a contracted state, the focal adhesion was changed, and the cytoskeleton was rearranged, which might be the causes of affecting cell adhesion ability.

On the other hand, changes in stem cell adhesion ability can determine its differentiation fate. In hMSCs, stimulated microgravity impaired the autophosphorylation of FAK and proline-rich tyrosine kinase 2 (PYK2), which might lead to reduced attachment and spreading of cells. Moreover, as the important positive regulator of FAK in osteogenic differentiation, microgravity inhibited osteogenesis through MAPK/ERK/Runx2 pathway [89]. Runx2 is an important transcription factor in osteoblastogenesis. With the reduced vinculin-containing focal adhesions, MSCs from HLU Rat exhibited decreased osteogenic potential in the osteogenic conditions with reduced transcriptional activity of Runx2 [88]. Besides MSCs, in adipose-derived stem cells (ADSCs), *CD44*, *β_1_ integrin*, *ColIII*, and *MMP1* were induced and cells performed an aggregated structure after 7 days of microgravity simulation. The up-regulation of CD44 might indicate the undifferentiated stage of ADSCs under simulated microgravity conditions [90]. So, we can infer that reduced cell adhesion capacity under microgravity conditions is important for stem cells to maintain their stemness and increase their potential for multi-directional differentiation.

Beyond that, other researchers have demonstrated that although microgravity destroys the adhesion ability of stem cells, it can also drive cell differentiation and transdifferentiation. Lu et al. demonstrated that space microgravity not only reduces adhesion molecule expression but also promote the hepatogenic differentiation of MSCs [87]. The upregulation of hepatocyte-specific cytokeratin 18 and albumin makes cell mature [87]. In addition, human lung cancer stem cells (NSCLCs were used) detached from the substrate and performed an apoptosis state after exposure to microgravity, leading to the loss of stemness features with decreased *Nanog* and *Oct4* genes [91]. Therefore, microgravity induces stem cell lineages to selectively differentiate into different phenotypes by changing their cell cycle. Furthermore, real microgravity experiments on SJ-10 satellite showed that the expression and activation of Runx2 were inhibited, and osteogenic differentiation was inhibited via the BMP2/SMAD and integrin/FAK/ERK pathways in hMSCs. Besides, adipogenic differentiation was promoted by increasing AKT and p38 MAPK activities [92]. Although microgravity inhibits stem cell adhesion, due to the diversity of cell types and functions, the effect of microgravity on stem cell differentiation is different. Hence, the molecular mechanism of stem cells’ response to microgravity is still needed to be further investigated (Table 5).

### 4.5. Microgravity Inhibits Osteoblast Adhesion and Differentiation

Astronauts often develop significant osteoporosis-like loss of bone mass, which has been attributed to microgravity. In the absence of gravity, there may be physiological changesin the cytoskeletal arrangement, adhesion, growth, and differentiation of bone cells [93]. The expression of adhesion molecules and ECMs in osteoblasts changed under the condition of microgravity. Kumei et al. reported that, compared to ground controls, the expression of osteopontin (OPN) was decreased in rat osteoblasts after exposing the cultures to microgravity in flight [94], which might lead to weakened cell adhesion ability because OPN promotes the attachment of cells to the bone surface in bone metabolism. On the other hand, stimulated microgravity increased the expression of OPN in human fetal hFOB 1.19 osteoblasts, and ECM such as laminin subunit α_1_, collagen type 1 α_1_, and fibronectin 1. However, these gene changes allowed the adhesive cells to detach from the surface and formed 3D structures [95]. Moreover, ex vivo experiment showed that simulated microgravity inhibits the expression of β_1_ integrin in human primary osteoblasts, and leads to a mesenchymal-like phenotype, indicating that osteogenesis is impaired [96]. Therefore, although microgravity has different regulatory effects on gene expression in osteoblasts derived from different species, it has the same effect on cell adhesion.

In addition, under the condition of microgravity, the number and structure of focal adhesions in osteoblasts were also changed. Exposure of primary osteoblasts and osteoblastic ROS 17/2.8 cells to microgravity reduced the number and average size of focal adhesions compared to control cells on the ground [93,97]. Furthermore, changes in the adhesion of osteoblasts affected the changes in the cytoskeleton and activation of intracellular signals, which determined the biological function of cells. Integrin-mediated cell adhesion in osteoblastic ROS 17/2.8 cells under microgravity was affected in parabolic flight and on a clinostat, and the changes were accompanied by disorganization of the cytoskeleton and disassembly of vinculin spots [98]. Actin filament rearrangement and focal adhesion formation might be regulated through the Rho GTPase signaling pathway [99]. The disrupted F-actin stress fibers and integrin signaling inhibited by focal adhesion kinase further inhibit osteoblastic differentiation [100]. Obviously, it is reasonable to think that the bone loss in weightlessness might arise from the repression of osteoblastogenesis via adhesion inhibition activation (Table 6).

### 4.6. Microgravity Regulates the Adhesion and Phenotype of Muscle Cells

During the space flight in the microgravity environment, zero gravity can have deleterious effects on muscle cells, especially skeletal muscle, including the expression changes of muscle growth/atrophy related genes, phenotype, and filaments structure changes of muscle cells, leading to arterial remodeling and muscle atrophy [101].

Recent studies have shown that multiple mechanisms are involved in microgravity-induced arterial remodeling. Kang et al. demonstrated that simulated microgravity disrupted the well-organized cytoskeleton in vascular smooth muscle cells (VSMCs), with reduced proliferation and promoted apoptosis [102]. Moreover, VSMCs changed from a synthetic phenotype to a contractile phenotype, with upregulation of smMHC expression [102]. In addition, with an in vivo experiment, the number of focal adhesions in the basilar artery was increased after 4-week HLU. The expression of p-FAK Y397 and p-Src Y418 was increased in SMCs of basilar arteries [103]. Activation of FAK combined with focal adhesions is an important event in early mechanical transduction and plays a critical role in vascular remodeling.

Additionally, in human skeletal muscle stem/progenitor cells (SMPCs) after 2 weeks of microgravity culture, the number of adherent cells was significantly reduced and cells formed into spheres with reduced numbers of myotubes. Moreover, the expression of Pax7 was also decreased in SMPC spheres through the TRAF6/ERK pathway [104]. Pax7 is a transcriptional factor critical for muscle development and regeneration [105]. Therefore, microgravity imbalanced the stability of SMPC pool, leading to insufficient muscle regeneration. Taken together, the adhesion of muscle cells plays an important role in regulating muscle morphogenesis. Thus, reduced adhesion of muscle cells might be responsible for the vascular remodeling and muscle atrophy under microgravity (Table 7).

### 4.7. The Function of Microgravity on Adhesion of Other Cell Types

There is increasing evidence that microgravity in space has many negative effects on nervous systems, visual function, as well as the skins of astronauts. The function of microgravity on the adhesion of cells in these organs has also been demonstrated.

Under simulated microgravity, the adhesion ability of primary cells from human brain nervous tissue was decreased, accompanied by highly disorganized β-tubulin structures in a circular pattern around the nucleus [106]. The cytoskeletal structure and distribution changes might be the main reason for changes in cell adhesion. Moreover, cells exhibited increased apoptosis, and it can be assumed that the function of the entire nervous system may be threatened by simulated or real microgravity. In addition, microgravity can also reduce the expression of β_1_ integrin, β_3_ integrin, and laminin subunit β-2 (LAMB2) in human adult retinal pigment epithelium cells [107]. As integrins are pivotal for focal adhesion formation and cytoskeleton rearrangements, this might explain the reason behind the effect of microgravity in promoting the detachment of cells detached from culture plate to form multicellular spheroids.

Furthermore, the expression of the cell–cell adhesion molecule E-cadherin in human keratinocytes was reduced during short exposure to microgravity. Mesenchymal markers such as α-SMA and vimentin were upregulated [108]. This means that the cell–cell adhesion of keratinocytes was attenuated, leading to epithelial-mesenchymal transition and invasive phenotypes in the microgravity environment. Consistent with these findings, in normal human dermal fibroblasts (NHDF), focal adhesion, cytoskeleton, and growth behavior were also changed by simulated microgravity through regulating integrin-β_1_ and E-cadherin [109]. Taken together, microgravity affects the physiological function of organs by regulating cell adhesion, and further regulating cytoskeletal rearrangement and phenotypic changes, etc., so that the human body can perceive and adapt to changes in the gravity environment (Table 8).

## 5. Conclusions and Perspectives

Microgravity is one of the major challenges facing astronauts during spaceflight. Exposure to microgravity has been associated with various significant physiological changes of the bone tissue as well as the cardiovascular, nervous, and immune systems, as well as pathological changes such as atherosclerotic plaques or even cancer. The migration, proliferation, differentation, and apoptosis of mammalian cells are affected by changes in cell adhesion during long-duration spaceflights. In this review, we have introduced the biological functions of cell–cell and cell–matrix adhesion and have summarized the roles of different adhesion molecules including integrins, Ig-SF, cadherins, and selectin in regulating cell adhesion. Based on the available literature, we have reviewed the effects of spaceflight and simulated microgravity on the adhesion capacity of endothelial cells, immune cells, cancer cells, stem cells, osteoblasts, muscle cells, nerve cells, etc.

Although several spaceflight and ground-based experiments have been performed to study the effects of microgravity on the adhesion ability of different cell types, the mechanisms by which microgravity affects cell adhesion are not fully understood and need to be further investigated. Firstly, it is still unclear how exactly cells sense microgravity and transduce signaling through adhesive protein complex. The major genes that are involved in intracellular regulation are still unknown. Therefore, it should be clarified if the changes of expression or changes of cellular localization of adhesion protein lead to the different cell adhesion behaviors. The localization and expression of adhesion-related intracellular key regulator protein can be furtherly detected by in situ hybridization. Moreover, it is unknown whether the effect and mechanism of ground-based microgravity on cell adhesion are the same as that of real microgravity in space. The earth-based simulation techniques such as using RPM or HLU cannot fully mimic the zero gravity of the spaceflight. For example, in tumor cells, the expression of ICAM1 protein and the performance of focal adhesion such as vinculin and cofilin are different between real and ground-based simulated microgravity [82]. The expression of E-cadherin and cell adhesion state in HUVEC and MCF-7 cells showed different under simulated microgravity and overloading condition [110]. In addition, bauer et al. used mass spectrometry and mRNA microarray to compare the different effect of microgravity on sialylation of adhesion proteins in FTC-133 thyroid cancer cells, MCF7 cells, and EA-hy926 cells [111]. Thus, transcriptomics, proteomics, and even single-cell sequencing approaches can be used to compare the genomic and epigenomic changes of cells in different microgravity scenarios, which can provide molecular biology information and mechanism of cell adhesion under microgravity.

Furthermore, based on the adverse effects of microgravity on cell adhesion and further adverse influence on human physiology, drugs that targeted on the regulators of cell adhesion need to be developed for disease prevention or treatment of astronauts during spaceflight. For example, Romswinkel et al. demonstrated that the key regulator tumor cell adhesion, *CXCR4*, was upregulated in spheroids of Ewing’s Sarcoma cells under simulated microgravity [112]. Therefore, the authors tried to use CXCR4 inhibitor to regulate the cell adhesion. However, it was found that inhibiting of CXCR4 did not change the size and number of spheroids [112]. This is because the transcriptional and post-translational modifications change the function of CXCR4. Therefore, small nucleic acid such as siRNA or miRNA which can directly target on and regulate the key effectors that mediate the mechanisms of cell adhesion should be studied. In addition, when screening targeted drugs, the selection of cell model that responds to simulated microgravity is also critical. It was reported that, compared with parental Jurkat cell line, the Jurkat/A4 cells are more sensitive to simulated microgravity with the changes of adhesion molecule ICAM-3 expression and cell morphology [113]. Therefore, Jurkat/A4 cells are useful models for studying microgravity effects and testing anti-cancer drugs. Cell adhesion is the basic determinant of biological development, and also one of the important influencing factors of diseases such as nerves and tumors [114,115]. Further studies should be undertaken to explore the effect and mechanism of regulators on cell adhesion in microgravity, especially for the development of new therapeutic targets and drugs that can greatly improve the health of astronauts on long space missions.

## Figures and Tables

**Table 1:** The summary of cell adhesion molecules.

Adhesion Molecules	Classification	Functions in Cell Adhesion	References
Integrins	24 known αβ-heterodimers, 18 α-subunits, and 8 β-subunits	Connection between the extracellular matrix (ECM) and the actin cytoskeleton of cells	[24]
Immunoglobulin superfamily (Ig-SF)	Intercellular adhesion molecule (ICAM-1)	Cell–cell adhesion by binding to specific ligands in the ECM and surrounding cells	[25]
	Vascular cell adhesion molecule (VCAM-1)	Leukocyte adhesion to endothelial cells	[26,27]
	Platelet endothelial cell adhesion molecule (PECAM-1)	The adhesion and accumulation of platelets	[28]
Cadherins	E-cadherins	Cell–cell adhesion in epithelial cells	[29]
	N-cadherins	Tumor intercellular adhesions	[30]
	VE-cadherins	Adhesive connections between endothelial cells	[31]
Selectin	E-selectin, L-selectin, P-selectin	Adhesion of platelets to tumor cells, lymphocyte homing, endothelial cells-tumor cells, interaction of tumor cells.	[32,33,34]

**Table 2:** Effects of microgravity on endothelial cells adhesion.

Cell/Mice Models	Mode of Microgravity	Relevant Changes	Mechanisms	References
HUVECs	Progress 40P mission	44 cell adhesion-related genes changed	/	[61]
EA.hy926	SJ-10 Satellite	ICAM-1 (-), VCAM-1 (-), CD44 (+)	/	[62]
EA.hy926	Random positioning machine (RPM)	Fibronectin (+), ICAM-1 (+), VCAM-1 (+)	IL-6 and IL-8 regulate adhesion molecules	[63]
Endothelial cells in the carotid artery of Rat	Hindlimb unweighted (HLU)	E-selectin (+), VCAM-1 (+)	/	[64]
Human umbilical vein endothelial cells (HUVECs)	Rotary cell culture system (RCCS)	Focal adhesions (-), actin fiber formation (-), apoptosis (+)	mTOR/Apaf-1 and miR-22 signaling pathway	[65]

**Table 3:** Effects of microgravity on immune cells adhesion.

Cell/Mice Models	Mode of Microgravity	Relevant Changes	Mechanisms	References
Human M1 macrophages	SpaceX CRS-3 mission	ICAM-1 (-)	Mechanically sensitive signals in the cell-polycarbonate binding region	[67]
Differentiated human U937 cells	2D clinostat, SIMBOX/Shenzhou-8 mission	ICAM-1 (+)	/	[68]
Rat	HLU	E-selectin (+), VCAM-1 (+), MCP-1(+), recruitment of monocyte to aortic endothelium	NF-κB pathway	[69,70]
Peripheral monocyte	Space missions	CD26L (-), HLA-DR (-)	/	[71]
Peripheral monocyte	Parabolic flight	Reduced peripheral monocyte adhere to ICAM-1	/	[72]

**Table 4:** Effects of microgravity on tumor cells adhesion.

Cell Models	Mode of Microgravity	Relevant Changes	Mechanisms	References
BL6-10 melanoma cells	RPM	Focal adhesions (paxillin and vinculin) (-), apoptosis (+)	FAK/RhoA-regulated mTORC1 and AMPK signaling pathway	[74,75]
U251 cells	RPM	Focal adhesions(-), cell viability and migration (-)	FAK/RhoA/Rock and FAK/Nek2 signaling pathway	[76]
MCF-7 cells	RPM	Focal adhesions(-), β1 integrin(-), β4 integrin (-)	Decreased kinases activity (such as FAK, PYK2, and ILK)	[77]
PC-3 cells	RPM	Adhesion (-), Talin1, Vinculin, and Cdh1 in multicellular spheroids (+)	/	[78]
Lung cancer cells line (CRL-5889)	RPM	Adhesion (-), spherical arrangement of the actin filaments, and apoptosis (+)	/	[79]
MCF-7 cells	TEXUS 54 rocket mission	E-cadherin (-), β1 integrin (-), actin arrangement	/	[80]
MCF-7 cells	RPM	E-cadherin (-), multicellular spheroids formation	E-cadherin autodegradation pathway	[81]
FTC-133 cell line	TEXUS-53 Mission	ICAM-1 (+), VCAM-1 (+), cofolin1(+)	/	[82]
FTC-133 cell line	RPM	ICAM-1 (-), cofolin1(-), disorganized vinculin	/	[82]

**Table 5:** Effects of microgravity on stem cells adhesion.

Cell Models	Mode of Microgravity	Relevant Changes	Mechanisms	References
hMSCs	RPM	VCAM-1^+^ cells (-), VCAM-1 (-), disrupted actin cytoskeleton, vinculin redistribution	/	[85,86]
Rat MSCs	SJ-10 Satellite	VCAM1 (-), ICAM1 (-), CD44 (-), vinculin (-), actin filaments depolymerization, hepatogenic differentiation (+)	Upregulataion of hepatocyte-specific cytokeratin 18 and albumin	[87]
Rat MSCs	HLU	Vinculin-containing focal adhesions (-), osteogenesis (-)	/	[88]
hMSCs	Rotating wall Vessel	Autophosphorylation of FAK and PYK2 (-), osteogenesis (-)	MAPK/ERK/Runx2 pathway	[89]
ADSCs	RPM	CD44 (+), β1 integrin (+), cell aggregation (+)	/	[90]
NSCLCs	RPM	Cell adhesion (-), stemness features (-)	Decreased Nanog and Oct4 genes	[91]

**Table 6:** Effects of microgravity on bone cells adhesion.

Cell Models	Mode of Microgravity	Relevant Changes	Mechanisms	References
Rat osteoblasts	Space flight	Cell adhesion (-)	Decreased osteopontin	[94]
hFOB 1.19 cells	RPM	Cell adhesion (-)	/	[95]
Human primary osteoblasts	RPM	Cell adhesion (-), mesenchymal-like phenotype	Decreased β_1_ integrin	[96]
Primary osteoblasts and Osteoblastic ROS cells	Foton M3 satellite	Focal adhesions (-)	Partly ERK proliferative-dependent pathway	[93,97]
Osteoblastic ROS cells	Parabolic flight and clinostat	Cytoskeleton disorganization, vinculin spots disassembly	β_1_ integrin-mediated	[98]
Human MG-63 cells	Foton M3 satellite	Number of focal contacts (-)	Rho GTPase signaling pathway	[99]

**Table 7:** Effects of microgravity on muscle cells adhesion.

Cell/Mice Models	Mode of Microgravity	Relevant Changes	Mechanisms	References
Vascular smooth muscle cells (VSMCs)	RPM	Cell adhesion (-), disrupted cytoskeleton, contractile phenotype	/	[102]
Rat	HLU	Focal adhesions number in a basilar artery (-)	Increased p-FAK Y397 and p-Src Y418	[103]
Skeletal muscle stem/progenitor cells (SMPCs)	Clinostat rotation system	Cell adhesion (-), myotubes number (-)	TRAF6/ERK pathway	[104]

**Table 8:** Effects of microgravity on adhesion of other cells.

Cell Models	Mode of Microgravity	Relevant Changes	Mechanisms	References
Primary cells from human brain nervous tissue	RPM	Cell adhesion (-), apoptosis (+)	Disorganized β-tubulin structures	[106]
Human adult retinal pigment epithelium cells	RPM	Cell adhesion (-)	Reduced expression of β_1_ and β_3_ integrin	[107]
Human keratinocytes	RPM	Cell adhesion (-), E-cadherin (-)	/	[108]
Normal human dermal fibroblasts (NHDF)	RPM	ECM proteins Adhesion molecules Cytoskeleton	Regulate of β_1_ integrin and E-cadherin	[109]

## Abbreviations

CAM	Cell adhesion molecules
ECM	Extracellular matrix
Ig-SF	Immunoglobulin superfamily
ISS	International space station
VE-cadherin	Vascular endothelial cadherin
FAK	Focal adhesion kinase
YAP	Yes-associated protein
ICAM	Intercellular adhesion molecule
VCAM	Vascular cell adhesion molecule
PECAM	Platelet endothelial cell adhesion molecule
EPCs	Endothelial progenitor cells
MSCs	Mesenchymal stem cells
FGFR	Fibroblast growth factor receptor
HUVEC	Human umbilical vein endothelial cells
RPM	Random positioning machine
HLU	Hindlimb unweighted
RCCS	Rotary cell culture system
MCP-1	Monocyte chemoattractant protein
HLA-DR	Major histocompatibility complex, class II, DR
PYK2	Proline-rich tyrosine kinase 2
ADSCs	Adipose-derived stem cells
OPN	Osteopontin
VSMCs	Vascular smooth muscle cells
SMPCs	Skeletal muscle stem/progenitor cells
LAMB2	Laminin subunit β-2
NHDF	Human dermal fibroblasts
